# A synthesis of youth activation initiatives and their impact on tuberculosis knowledge, knowledge-seeking, and healthcare-seeking behavior in vulnerable populations

**DOI:** 10.3389/ftubr.2025.1668711

**Published:** 2026-01-09

**Authors:** Ana-Maria Ionescu, Joelle Mak, Qu Yan, Nishant Kumar, Raghuram Rao, Sanjay Mattoo, Deepak Balasubramanian, Rachana Acharya, Subhi Quraishi, Niraj Sharma, Kep Nurliyanti, Juliana Chin, Siva Anggita, Tiffany Pakasi, Sarah Rahma, Ingrid Eshun-Wilsonova

**Affiliations:** 1 Johnson & Johnson, New Brunswick, NJ, United States; 2 London School of Hygiene & Tropical Medicine (LSHTM), London, United Kingdom; 3 MTV Staying Alive Foundation, London, United Kingdom; 4 National Center for Tuberculosis Control and Prevention, Chinese Center for Disease Control and Prevention, Beijing, China; 5 National Tuberculosis Elimination Program (NTEP), New Delhi, India; 6 PATH India Office, Seattle, WA, United States; 7 ZMQ Development, New Delhi, India; 8 Stop TB Partnership Indonesia, Jakarta, Indonesia; 9 Indonesia Muda Untuk Tuberkulosis, Jakarta, Indonesia; 10 Sub-Directorate of Tuberculosis, Ministry of Health of Indonesia, Jakarta, Indonesia; 11 Department of Occupational Safety and Health, Faculty of Public Health, Universitas Airlangga, Surabaya, Indonesia

**Keywords:** youth activation, health promotion, tuberculosis, health-seeking behavior, knowledge-seeking behavior

## Abstract

**Background:**

Annually, up to 3 million people with tuberculosis (TB) fail to receive care due to delays in seeking TB care. Alongside active case finding, identifying cost-effective strategies that successfully mobilize vulnerable populations, such as young adults, to proactively present to care early is critical for ending the TB epidemic. Enabling populations to achieve optimized knowledge levels and proactively self-present to care may be more efficient than population-wide screening.

**Methods:**

Between August 2021 and December 2023, five youth activation initiatives (“Be the Change”, MumbraTB-ACTS, and MTV Nishedh in India; TB Warriors in Indonesia; UVTB5+ in China) were implemented. Initiatives were assessed on reach and program engagement, TB knowledge improvement, knowledge-seeking behaviors, healthcare-seeking behaviors, TB volunteering, and TB self-screening, as precursors to presentation to care. Program outcomes measurements included number of individuals reached and knowledge change before and after implementation using knowledge, attitudes, and practices (KAP) surveys. Core healthcare-seeking behavior indicators were also tracked where feasible.

**Results:**

Across programs, various strategies were implemented to reach youth including social media, hybrid case-finding, mass media, gamification and peer education, and/or volunteer-driven health promotion. Variability in measurement and reporting of program outcomes confounded the synthesis of information across initiatives, but collectively the initiatives resulted in broad reach within each local context, resulting in more than 200 million youths reached and 100 million engaged. More than 800,000 individuals reported knowledge-seeking activities with >50,000 completed KAP surveys. Initiatives demonstrated evidence of empowering communities to proactively undertake screening and/or volunteer for TB initiatives. Three also measured positive improvements in knowledge of disease transmission, symptoms, and curability.

**Conclusion:**

These data highlight several locally successful strategies for increasing TB awareness and knowledge, and encouraging TB care seeking, as well as mobilizing youth to volunteer as community TB KAP advocates. Next steps to understanding the true impact of such activation TB initiatives should include the development of a global framework to provide guidance on best practices for impact assessment, particularly at the healthcare-seeking stage. This could support healthcare workers in their provision of equitable access to quality care and implementation of TB initiatives.

## 1 Introduction

Tuberculosis (TB), which is caused by the bacillus *Mycobacterium tuberculosis* and transmitted via droplet infection, was estimated at 10.8 million new cases globally in 2023, likely surpassing COVID-19 as the leading cause of death by a single agent (1). The burden is highest in India (27%), Indonesia (10%), and China (7%) (1). While TB is usually preventable and curable, without treatment the mortality rate is high (50%) (1). Only 8.2 million cases were reported as newly diagnosed in 2023, suggesting that close to 3 million people were living with undiagnosed TB in the community (1), and consequently failed to receive access to quality care (2).

Patient delay in seeking care for TB symptoms is the most important and largest contributor to the time lag experienced by patients within the cascade of care, from awareness of first symptom onset to initiation of treatment (3, 4). Factors such as TB-associated stigma, misconceptions about disease severity, and limited access to healthcare can greatly impact patient delay (5, 6). For example, the National TB Prevalence Survey in India (2019–2021) found that a majority (64%) of the symptomatic population did not seek healthcare services (7). However, patient delay can be addressed by either enhancing patient-initiated healthcare seeking and/or improving provider-initiated pathways to diagnosis; both approaches also require community acceptance (8–10). Both approaches are also needed to end the TB epidemic, and it is possible that, over the long term, increasing TB knowledge levels and proactive self-presentation to care, may be more efficient than population-wide screening.

To change behavior, there is a need to address TB stigma through education to improve knowledge, attitudes, and practices (KAP) (11–17). Previous studies have shown increased TB knowledge and treatment-seeking behaviors after exposure to TB awareness campaigns (9, 10, 18). Healthcare workers in many countries with a high burden of TB have implemented various initiatives to reach at-risk populations, expand TB knowledge, and recruit volunteers to promote healthcare-seeking behaviors and improve access to treatment (19–23).

The incidence and transmission of TB is high among teenagers and young adults in India, Indonesia, and China (1, 24–27), especially in high-density settings, making them an important group for implementing initiatives focused on encouraging early and prompt presentation to care (28, 29). Given that nearly three-quarters of young people globally are online and many are actively engaged in social causes (30, 31), bespoke digital health promotion, particularly when tailored to local sociocultural and economic contexts, can be beneficial (5, 32, 33).

To explore the impact of such active engagement of youth for TB, five private sector-supported youth activation and education initiatives in high-prevalence countries (India, Indonesia, and China) have adopted pragmatic methods, including digital content, to empower and equip young people to become advocates for their own health and for those in their communities. These initiatives aimed to increase TB knowledge, build a cadre of youth changemakers, and improve healthcare-seeking behavior. In this paper, we review lessons learned from these initiatives to inform the development of future programs.

## 2 Materials and methods

### 2.1 Program descriptions

Five youth activation and education initiatives (three in India, one in China, and one in Indonesia), all supported through the private sector (e.g., provision of funding, design co-creation, technical assistance), were implemented to impact early presentation to TB care and to help end TB. These initiatives took place around the same time period (2021–2023) and targeted similar demographics, specifically young people across various Asian regions with a relatively high burden of TB. The duration, target populations, objectives, tactics, and geographical coverage of each initiative are detailed in Table 1.

**Table 1:** Description of the five youth activation initiatives evaluated in this study.

**Campaign**	**Implementers**	**Country, population**	**Target population**	**Format/components**	**TB-related aims**	**Link to report**
“Be the Change” (March to December 2022)	Digital content agency Digital media agency	India, 1.4 billion	Young adults (18–34 years) living in eight cities^a^	•Digital media advertising campaign (with precision targeting through social media, using music/rap anthem as message medium and youth icons as the messengers); social media amplification partnerships •Survey to identify a high-intent audience using digital footprint to determine target persona	To promote health-seeking behaviors, share information, and recruit potential TB changemakers	Impact Report available at: https://www.path.org/our-impact/resources/learnings-from-innovative-approaches-to-tb-case-finding-and-youth-activation-in-india-and-indonesia/ Direct download link: https://media.path.org/documents/9_JJ_Be_the_Change_for_TB-Brochure-logo_updated_13_Dec2024_V2.pdf
MumbraTB-ACTS (August 2021 to January 2023)	ZMQ Development	India, 1.4 billion	Residents of urban slums in the Mumbra-Kausa area of Thane	•Hybrid program using digital tools, house-to-house screening, and community activities •Self-screening toolkits promoted through billboards, social media, and health centers	To strengthen active case-finding through community engagement in urban slums and low-literacy areas	Impact Report available at: https://zmqdev.org/zmq/project-reports/
MTV Nishedh S2 (November 2022 to June 2023)	MTV Staying Alive Foundation	India, 1.4 billion	Adolescents and young adults (16–25 years)^b^, in Bihar, Maharashtra, Uttar Pradesh states	•Digital edutainment drama series (10 episodes, approximately 20 min in length); multi-platform distribution and parallel social media “promotion buzz” •Before and after surveys among exposed and unexposed participants to track knowledge-seeking and healthcare-seeking behavior change	To reduce stigma associated with TB and to encourage testing, reduce transmission, and improve treatment and knowledge	Impact Report available at https://www.mtvstayingalive.org/impact/ Direct download link: https://cdn.sanity.io/files/5lp3a2r6/production/cb792474ed468d478c694b8086aa6ae198d92ac8.pdf
TB Warriors (March 2022 to December 2023)	Digital content agency Digital media agency STOP TB Partnership Indonesia; Indonesia Muda Untuk Tuberkulosis (IMUT)	Indonesia, 280 million	Adolescents and young adults; college students; four cities^c^	•Y1 Social media campaign (two waves, each 3 months), directing to website/online game •Y2 Multi-platform campaign—evolved social media campaign 3 months (including intent survey to engage potential volunteers social media amplification partnerships) and onsite volunteer-based campaign at three universities involving educational talk shows and informational booths	To raise TB awareness in college students, expand screening, and link young people to care	Impact Report available at https://www.stoptbindonesia.org/kampanye-digital Direct download link: bit.ly/FinalReportTBW
UVTB5+ (March 2022 to December 2022)	China CDC^d^ and IHECC	China, 1.4 billion	First- and second-year college students at 20 universities each in Zhejiang and Sichuan provinces	•Volunteer-led campus campaign including onsite screening •Online and offline educational activities, such as knowledge contests, orientation sessions for new students, and disease awareness activities	To increase TB awareness among students, encourage health-seeking behaviors, and enhance early diagnosis	Impact Report available at https://tb.chinacdc.cn/2024zxdt/202405/t20240527_278939.html Direct download link: https://tb.chinacdc.cn/2024zxdt/202405/P020250519546902538937.docx

Johnson & Johnson provided technical assistance to all the programs.

^a^Ahmedabad, Bangalore, Chennai, Delhi, Hyderabad, Lucknow, Mumbai, and Patna.

^b^Those living in urban and peri-urban areas with access to television (cable/dish) and the internet in the Bihar, Maharashtra, and Uttar Pradesh districts were targeted by mass media.

^c^Jakarta, Yogyakarta, Surabhaya, Bali.

^d^Tuberculosis Control and Prevention Center.

CDC, Center for Disease Control and Prevention; IHECC, International Health Exchange and Cooperation Center, National Health Commission; IMUT, Indonesia Muda Untuk Tuberkulosis; TB, tuberculosis.

“Be the Change” (India) utilized social media to promote healthcare-seeking behaviors and recruit youth volunteers as potential changemakers. MumbraTB-ACTS (India) implemented a hybrid format to active case-finding in urban slums by combining house-to-house visits with community health promotion and self-screening tools. MTV Nishedh Season 2 (India) created a digital edutainment series (for multiple health issues, evolved from a Season 1 mass media edutainment initiative) with the TB components focused on reducing TB stigma (particularly experienced by female patients), improving awareness of symptoms and transmission, while encouraging testing and treatment.

TB Warriors (Indonesia) used online health promotion to raise disease awareness and lead the audience to a gamified website that gauged knowledge- and healthcare-seeking behaviors, including online-self screening. Volunteer-based onsite university campus screening drives were also conducted.

UVTB5+ (China) also aimed to increase university student TB knowledge on university campuses, encouraging health-seeking behaviors and early testing through trained peer volunteers.

All programs primarily sought to enhance patient-initiated pathways for seeking TB care. These initiatives did not require local ethical approval.

### 2.2 Program measures and definitions

The five initiatives (Table 1) were evaluated to assess six key goals; each initiative used at least one online platform (Table 2). Although each of the initiatives were assessed differently, there were commonalities that allowed assessment of (1) reach and engagement, (2) change in KAP, (3) extent of knowledge-seeking behaviors, (4) healthcare-seeking behaviors, (5) volunteer recruitment and engagement, and subsequently (where measured) (6) impact on TB screening (Table 3). We evaluated the impact of all five initiatives in reaching their target audience and assessed reported changes in TB KAP post-initiative. In addition, we explored how each initiative promoted knowledge-seeking behaviors and motivated healthcare-seeking behaviors. The precise definitions of the six key goals used in evaluating each initiative varied and are given in Table 3.

**Table 2:** Summary of online platforms used and link to content for initiatives evaluated.

	**Be the Change**	**MumbraTB-ACTS**	**MTV Nishedh S2**	**TB Warriors Y2**	**UVTB5+**
Digital platforms/ad type	Banner ads on Google Ads and Google DV360 Responsive search ads on Google Ads Google Ads search campaign Facebook/Instagram/YouTube ads Website for TB knowledge and volunteer enrollment	Digital apps	Short-form digital videos MTV channel MTV Nishedh and partners Voot, Colors Rishtey Social media handles (YouTube, Facebook, web) Other video on demand streaming platforms	Banner ads on Google Ads and Google DV360 Responsive search ads on Google Ads Google Ads search campaign Website for TB knowledge and self-screening Social media handles (Instagram, Facebook, TikTok) Communication platform (WhatsApp) On-campus website/system information	On-campus websites WeChat public accounts
Content/further description links	https://www.instagram.com/bethechangefortb/	https://yourstoryteller.org/tbstoryteller/	https://www.youtube.com/@mtvnishedh	https://www.instagram.com/warriors.tb/	https://tb.chinacdc.cn/zxdtx/202311/t20231123_270831.html

TB, tuberculosis.

**Table 3:** Summary of outcome measures and definitions for initiatives evaluated.

**Campaign**	**Outcome measures**					
	**Impresssions**,^a^ **reach**,^b^ **and engagement**^c^	**KAP**	**Knowledge-seeking behavior** ^d^	**Healthcare-seeking behavior** ^e^	**Volunteer engagement**	**Screening and diagnosis**
“Be the Change”	•Online number of unique individuals who see campaign content (reach) •Ad clicks and website visitors (engagement)	•Pre- and post-survey answers	•Education-related content clicks •Search engine volume change	•National TB helpline clicks •Downloads of government digital self-screening app	•Volunteer sign-ups	•NA (data from self-screening app downloads could not be linked to actual number of self-screenings undertaken)
MumbraTB-ACTS	•Onsite number of unique individuals who see campaign content (reach) •Individuals exposed to poster campaign (reach)	•Baseline knowledge survey answers	•Participation in active ground building (community sessions using digital, school-based awareness session, storytelling sessions, m-learning)	•NA	•NA	•Digital self-screening •Parallel active case finding door-to-door and community locations (individuals screened and TB cases identified)
MTV Nishedh S2	•Number of unique individuals who see campaign content (reach) •Video views (engagement) •Social media interactions (engagement)	•Pre- and post-survey answers •Interviews/focus group discussions	•Interactions on social media •Completed episode views	•National TB helpline clicks and call confirmations	•NA	•Question on intent to screen pre- and post-intervention
TB Warriors	•Number of unique individuals who see campaign content, online and onsite (reach) •Ad clicks and website visitors (engagement)	•Baseline intent survey answers	•Education-related clicks including playing games and competitions •Search engine volume change •Onsite educational talk attendance	•Clinic searches •Online self-screening	•Survey on willingness to volunteer	•Digital self-screening (individuals screened)
UVTB5+	•Onsite number of unique individuals who see campaign content (reach) •Onsite Live events attendees (health education lectures, information desks, free onsite clinics run by hospital staff, campus display boards; reach)	•Pre- and post-survey answers	•Online science popularization “reads” •Offline knowledge competitions attendees •Innovative/creative activities attendees	•NA	•Actual volunteers	•Post-intervention campus-wide active case finding (individuals screened)

Impressions: number of times content is shown to users; Reach: number of (unique) users who see the content, even if they see the content multiple times; Engagement: number of likes, shares, comments, website visitors, video views, poster views; Knowledge-seeking behavior: participation in educational activities whether virtual (information searches, clicks for knowledge, plays game, takes quiz, completes survey, direct messaging, completed videos) or face-to-face (lectures/talks, storytelling, information stations); Healthcare-seeking behavior: volunteering including peer education, helpline number, clinic search, screening offline to online.

^a^Times content is shown to users.

^b^Unique individuals who see campaign content.

^c^Interactions beyond seeing content.

^d^Deeper level of engagement with content such as participation in prolonged educational activities whether virtual or face-to-face.

^e^Helpline number reach-outs and local clinic search.

KAP, knowledge, attitudes, and practices; NA, not available; TB, tuberculosis.

“Impressions” were the number of times content is shown to users, “reach” was defined as the number of unique users who viewed content (even if the content was viewed multiple times), and “engagement” was defined as the total number of times unique users subsequently interacted with content beyond merely seeing it (e.g., watching videos; number of likes, shares, comments; opening linked content [website pages, videos, posters], shares, comments, etc.; Table 3, footnote). Reach as a percentage was calculated as the number of (unique) users who saw the content divided by the number of impressions (number of times content was shown to users). Engagement as a percentage of reach was estimated by dividing the number of individuals with documented engagement by the number reached by that initiative. “Knowledge-seeking behavior” was defined as actions representing a deeper level of engagement in prolonged education-building activities whether virtual (information searches, accessing and interacting with knowledge-building content beyond initial engagement, game content, playing informational games, participating in TB knowledge quizzes, direct messaging, watching educational videos in their entirety) or face-to-face (attendance at lectures, talks, storytelling sessions, visiting information stations). “Healthcare-seeking behavior” was defined as actions closest to individuals taking action to seek care (calling TB helpline numbers and undertaking searches to identify location of closest local TB clinic). We also evaluated volunteer recruitment as a source of healthcare worker (HCW) support in their health promotion responsibilities in “Be the Change,” TB Warriors, and UVTB5+. Finally, in three of the five programs, participant journey was tracked through to “screening and diagnosis.” TB-related behavioral changes as a percentage were calculated as the number of knowledge-seeking behaviors, healthcare-seeking behaviors, or volunteering as proportions of those showing engagement.

Built-in online analytics were used to capture the data from each of the digital platforms deployed across the five initiatives (those listed in Table 2). Actual engagement with clinics or healthcare support systems following changes in healthcare-seeking behaviors was not assessed across initiatives.

Below, we describe how each of the five initiatives operationalized the measures.

1) “Be the Change” (India)

An online pre-campaign survey targeted the best-performing cohorts from an initial intent survey and assessed their basic awareness of TB (Supplementary Table S1). Using digital research to identify potential volunteers, targeted social media campaigns and a rap song were then launched to normalize TB conversations. A post-campaign survey assessed changes in knowledge and behaviors. Available outcomes included correct survey answers before and after the campaign, TB education clicks, national TB helpline clicks, downloads of a government TB screening app, and volunteer sign-ups.

2) MumbraTB-ACTS (India)

A baseline survey was administered to 90 randomly selected community youth members to understand demographics, health infrastructure, and existing TB knowledge. Face-to-face sessions, digital tools, and poster campaigns were then used to increase awareness while house-to-house and community event screening took place. Behavior outcomes included baseline survey results and number of people engaging in knowledge-building activities. Numbers of households identified, people screened, presumptive TB cases, participants tested, and positive TB cases were also recorded.

3) MTV Nishedh (India)

Telephone baseline surveys were conducted before (November 2022) and after (March 2023) the broadcast of the ten-episode Nishedh Season 2 edu-series in the states of Bihar, Uttar Pradesh, and Maharashtra (Supplementary Table S2). These regions have relatively low literacy levels and have a significant population living in urban slums with high TB burden. Participants were classified as exposed (watched ≥2 episodes) or unexposed (watched ≤ 1 episode). As watching episodes required a sustained time commitment, participants classified as exposed were counted as having demonstrated engagement. Outcomes included knowledge and attitude shifts from the surveys, interview and focus group findings, and clicks to videos, educational content, and the national TB helpline (shown at the end of each episode and promoted through social media).

4) TB Warriors (Indonesia)

Both the 1.0 online campaign in 2022 and the 2.0 online campaign in 2023 engaged participants of the TB Warriors online game. The 2.0 onsite campaign was held from June to December 2023 at four Indonesian universities. Prior to the campaign, a digital survey targeting people aged 18–34 years was conducted to identify and engage potential volunteers (Supplementary Table S3). The survey results were used to identify the ideal setting (universities) to recruit volunteers (TB Warrior Champions) based on the ideal candidate profile. Recorded outcomes were online engagement with campaign-related content (e.g., playing the game), survey answers on awareness and willingness to volunteer, education-related clicks, self-screening, clinic searches, and individuals screened.

5) UVTB5+ (China)

TB awareness among first- and second-year college students was assessed using an electronic questionnaire before and after educational and screening activities. Eight core knowledge questions were administered digitally to randomly selected students (Supplementary Table S4). The total awareness rate was calculated by dividing the total number of correct answers by the total number of answers from all participants. The awareness rate of each item was the percentage of participants who answered the question correctly. In addition to the knowledge survey, outcomes included online educational “reads,” onsite activity attendance, number of volunteers, and individuals screened.

### 2.3 Statistical analyses

Given the substantial heterogeneity in design and outcomes across the five initiatives, statistical analyses were not conducted. All data are presented descriptively.

## 3 Results

Information on demographics for each initiative is summarized in Table 4. The targeted population included vulnerable youth cohorts from lower fiftieth percentile household income (“Be the Change” and MumbraTB-ACTS). There was an even split between sexes, although there were more female participants in “Be the Change” and more male participants in TB Warriors. A diversity of approaches and settings of the initiatives was used (e.g., knowledge seeking vs. healthcare seeking, volunteering; both online and offline; Table 4).

**Table 4:** Target audience demographic data for initiatives evaluated.

	**Be the Change**	**MumbraTB-ACTS**	**MTV Nishedh S2**	**TB Warriors Y2**	**UVTB5+**
Target audience	Lower 50% household income	Lower 50% household income	16–25 years	18–34 years	18–20 years
Demographics, *n* Completed surveys	Intent survey 421,000 responses	90	697 exposed (>2 episodes viewed), 661 unexposed ( ≤ 1 episode viewed)^a^	15,639	4,414
Age categories	18–24 years: 85%	NA	16–18 years: 36%	18–24 years: 84%	< 20 years: 34%
	25–34 years: 15%	NA	19–25 years: 64%	25–34 years: 16%	≥20 years: 66%
**Sex**					
Male	44%	NA	48%	68%	52%
Female	56%	NA	52%	32%	48%
**Income**					
Lower	40%	NA	NA	22%	40%
Higher	60%	NA	NA	78%	60%

^a^An unaware/don't remember group (*n* = 536) was also counted but not included in the analysis.

NA, not available; TB, tuberculosis.

### 3.1 Reach and engagement

Initiatives measured their digital impact cascade through “impressions” (number of times campaign content was shown), “reach” (unique individuals who viewed campaign content), and “engagement” (subsequent interactions with content beyond merely viewing it). Levels of engagement ranged from 4% of reach (1.95 million for “Be the Change”), to 16% of reach (approximately 2 million for TB Warriors), to 77% of reach (MTV Nishedh Season 2, with over 130 million digital streaming and social media views and over 3 million television viewers). In China, UVTB5+ engaged more than 600,000 on-campus participants representing 90% of overall reached population; a relatively similar contained population was targeted in MumbraTB-ACTS in India, which focused on a distinct urban slum geography with initiative reaching the entire 400,000 population living there (Table 5). Within multi-year initiatives (TB Warriors and MTV Nishedh), a learning loop took place in which lessons from the previous year were incorporated into the next year. We assume that this contributed to the almost doubling of healthcare-seeking actions from total knowledge and healthcare seeking, due to progressive fine-tuning of messaging and targeting.

**Table 5:** Impact on TB-related behavior for initiatives evaluated, 2022–23.

***N*, number of people**	**Be the Change**	**MumbraTB-ACTS**	**MTV Nishedh S2**	**TB Warriors**	**UVTB5+**
Impressions^a^	349,000,000	NA	2,000,819,000	371,006,112	NA
Reach^b^ (%)^c^	55,000,000 (16%)	400,000 (NA)	133,270,000 (7%)	12,765,110 (3%)	680,000 (NA)
Engagement^d^ (%)^e^	1,950,000 (4%)	400,000 (100%)	103,018,700 (77%)^f^	2,003,771 (16%)	615,039 (90%)
**Impact on TB-related behavior**					
Knowledge-seeking^g^ (%)^h^	13,000 (0.6%)	93,943 (23%)	183,296 (0.2%)	77,983 (4%)	443,451 (72%)
Healthcare-seeking^i^ (%)^h^	106,153 (5%)	NA	11,500 (0.01%)	7,215 (0.4%)	NA
Volunteering (%)^h^	35,938 (2%)	NA	NA	3,933 (0.2%)	11,309 (1.8%)
Screening^j^	NA	180,930	12 colleges; ~1,000	19,704	617,025

^a^Impressions: number of times content is shown to users.

^b^Reach: number of (unique) users who see the content, even if they see the content multiple times—unique individuals who see campaign content.

^c^Percent reach calculated as number of (unique) users who see the content divided by impressions (number of times content is shown to users).

^d^Engagement: number of likes, shares, comments, website visitors, video views, poster views—interactions beyond seeing content.

^e^Percent engagement calculated as number of interactions beyond seeing content divided by number of (unique) users who see the content.

^f^Participants classified as exposed were counted as having demonstrated engagement.

^g^Knowledge-seeking behavior: participation in educational activities whether virtual (information searches, clicks for knowledge, plays game, takes quiz, completes survey, direct messaging, completed videos) or face-to-face (lectures/talks, storytelling, information stations)— deeper level of engagement with content such as participation in prolonged educational activities whether virtual or face-to-face.

^h^Percent TB-related behaviors were calculated as the number of knowledge-seeking behaviors, healthcare-seeking behaviors, or volunteering as proportions of those showing engagement (i.e., interactions beyond seeing content—number of likes, shares, comments, website visitors, video views, poster views).

^i^Healthcare-seeking behavior: volunteering including peer education, helpline number, clinic search, screening offline to online, including helpline number reach-outs and local clinic search.

^j^Screening across initiatives consisted of online self-screening, onsite campus screening, house-to-house level screening, and community-level screening as described in Table 1.

NA, not assessed; TB, tuberculosis.

### 3.2 Changes in TB KAP (knowledge and attitudes components)

All initiatives included digital surveys (with the number of responses varying from thousands through to tens of thousands), providing solid data for quantitative analyses. Most participants completing surveys were 18–25 years of age. Table 6 highlights pre- vs. post-initiative changes or exposed vs. non-exposed target population KAP changes.

**Table 6:** Change in knowledge, attitudes, and practice based on baseline and post-campaign surveys across initiatives.

**Select KAP questions**	**Be the Change** ^**a**^				**MumbraTB-ACTS** ^**b**^				**MTV Nishedh S2** ^**c**^				**TB Warriors** ^**d**^				**UVTB5**+^**e**^			
	**Q#**	**Pre**	**Post**	**Change**	**Q#**	**Pre**	**Post**	**Change**	**Q#**	**Not exposed**	**Exposed**	**Change**	**Q#**	**Pre**	**Post**	**Change**	**Q#**	**No health education**	**With health education**	**Change**
**Knowledge**																				
Disease awareness	#1	74%	–	NA		32%	–	NA		–	–	NA		64%	–	NA		–	–	NA
Transmission		67%	70%	4%		30%	–	NA		40%	74%	84%		–	–	NA		82%	94%	15%
Symptoms		11%	12%	9%		41%	–	NA		82%	90%	9%		–	–	NA		84%	93%	11%
Curability		82%	84%	2%		–	–	NA		90%	97%	8%		–	–	NA		61%	77%	26%
**Attitudes (discriminatory attitude)**																				
TB as “disease of the poor”		63%	63%	0%		–	–	NA		56%	65%	16%		–	–	NA		–	–	NA
Discomfort in engaging with TB patients		–	–	NA		–	–	NA		75%	84%	12%		–	–	NA		11%		NA
**Practice (knowledge-seeking behavior)**																				
Willing to seek information		–	–	NA		–	–	NA		14%	25%	79%		–	–	NA		84%	94%	12%
Willing to share a positive TB test		–	–	NA		–	–	NA		41%	55%	14%		–	–	NA		–	–	NA
**Practice (healthcare-seeking behavior)**																				
Support resources		52%	55%	6%		–	–	NA		10%	20%	100%		–	–	NA		82%	86%	5%
Willing to get tested		–	–	NA		–	–	NA		43%	63%	47%		–	–	NA		81%	91%	12%
Willing to volunteer		32%	–	NA		–	–	NA		–	–	NA		68%	–	NA		72%	86%	18%

^a^Participants: Pre *n* = 4,892 and post *n* = 4,794 for TB as “disease of the poor,” transmission, symptoms, curability, and support resources rows; *n* = 24,712 for disease awareness and willing to volunteer rows.

^b^Participants: *N* = 90.

^c^Participants: *N* = 1,894 for all rows; exposed *n* = 697, not exposed *n* = 661, did not know or could not say number of episodes watched *n* = 536.

^d^Participants: *n* = 3,416 for disease awareness and willing to volunteer rows.

^e^Participants: No health education *n* = 2,973 and with health education *n* = 14,518 for transmission, symptoms, curability, willing to get tested, willing to seek information, and willing to volunteer rows; *n* = 17,491 for discomfort in engaging with TB patients row. Change (%) captures the proportionate change value comparing post- to pre-initiative values = (Post-Pre/Pre) × 100. Percentages are calculated on data; all values in tables are rounded to whole percentages.

KAP, knowledge, attitudes, and practices; NA, not available; TB, tuberculosis.

In “Be the Change,” there was a 30% increase in the number of respondents who “correctly” completed the whole survey (from 3%; *n* = 134 pre-campaign to 4%; *n* = 171 post-campaign; questions covering intent and knowledge; shown in Supplementary Table S1). Modest improvements (2%−9% change) in awareness of disease transmission, symptoms, curability, and awareness of resources, such as Directly Observed Treatment Short-Course (DOTS) treatment centers were captured; however, no change was observed in the perception that TB only occurs in lower socioeconomic groups. There was a 3% increase in the percentage of overall correct responses, from 57 to 60% pre- vs. post-campaign. Males saw a 9% increase (from 53 to 58%) while females' awareness remained relatively unchanged, albeit at a higher starting level (60−61%).

Exposure to MTV Nishedh was also positively associated with knowing more about TB transmission (from 40 to 74%; 85% change), symptoms (from 82 to 90%; 10% change), and curability (from 90 to 97%; 8% change; Table 6). Of note, while only 69% of participants knew at baseline that TB treatment was free in India, 90% were aware at endline—this represents a particularly important 30% change for the lower socioeconomic strata, as people with TB symptoms seeking care report having equal preference in choosing between public and private sector—even though there is documentation that standard TB care in private sector is not only more expensive but can also be less effective than the one provided in public sector (20, 34). Surprisingly, the survey results suggest that the campaign may not have diminished potentially discriminatory attitudes, with the percentage of participants reporting feeling uncomfortable in the presence of a person with TB increasing from 75% at baseline to 84% at endline (12% change). Also, Season 2 campaign-exposed participants were less willing to share a TB diagnosis with another person than unexposed participants (55 vs. 41%)—this was in stark contrast to previously reported assessments from Season 1 peer education programs (conducted in selected provinces of the Rajasthan district during August to December 2020), where participants reported becoming more comfortable with being close to someone with TB (increase from 59 to 66%; 12% change) and not feeling the need to keep a family member's diagnosis secret (increase from 48 to 64%; 33% change).

Similarly, UVTB5+ initiative-related student responses indicated a modestly increased TB knowledge, albeit from a high baseline (Table 6). Participants achieved a 26% notable difference in those who knew disease was curable (61−77%) between those attending health education and those not attending. Despite this, 11% of participants still had a discriminatory attitude toward people with TB (based on responses to “If someone around you gets tuberculosis, what is your attitude toward him/her?”).

While comparative pre- and post-campaign data are not available for MumbraTB-ACTS or TB Warriors, the baseline evaluation informing campaign messaging found that only 32 and 64%, respectively, of respondents reported knowing about TB and only 30% in MumbraTB-ACTS knew about transmission.

### 3.3 Knowledge-seeking behavior

**Figure 1:**
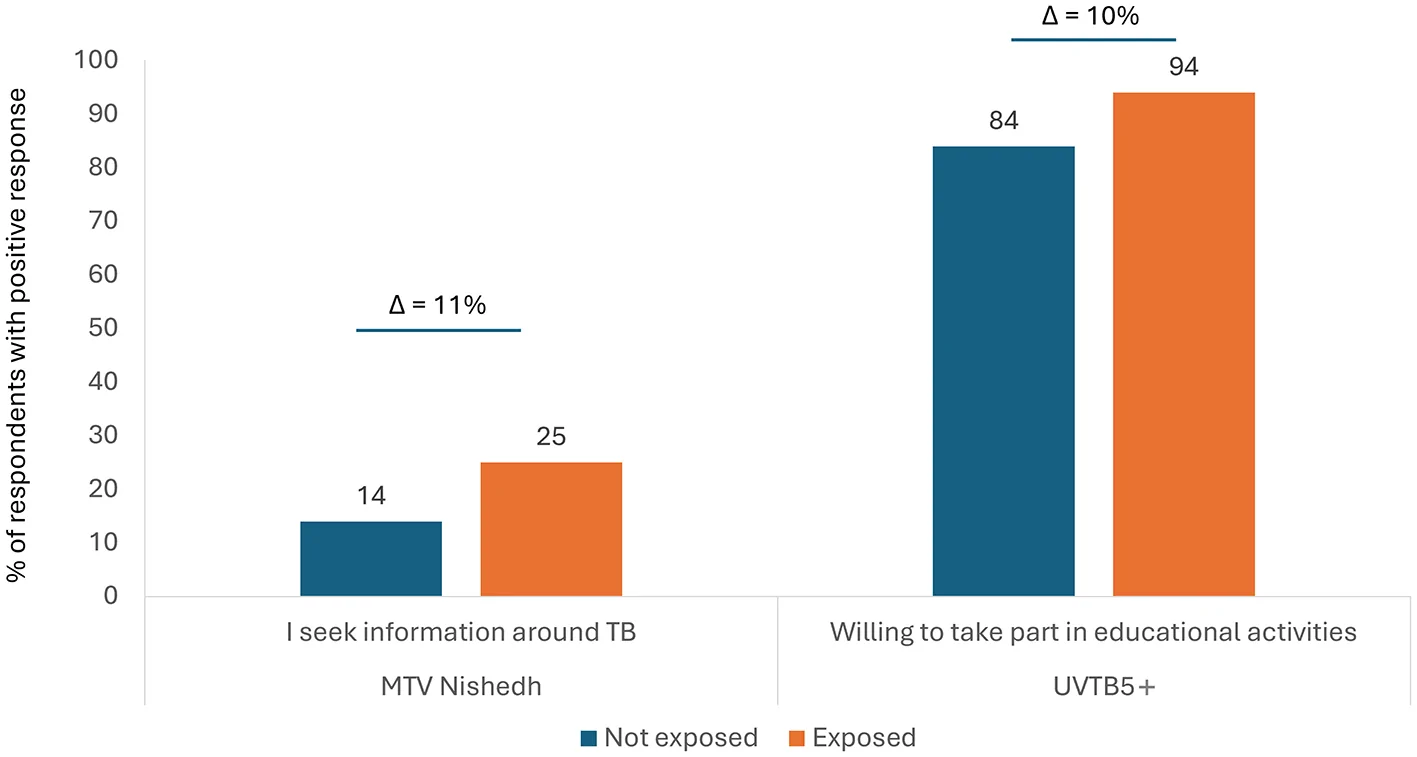
Knowledge-seeking behaviors. Responses on seeking TB information in MTV Nishedh and UVTB5+ initiatives in those not exposed to the initiative and exposed populations. TB, tuberculosis.

Information on knowledge-seeking behavior is available from surveys used in MTV Nishedh and UVTB5+. After watching MTV Nishedh, 25% of participants sought information about TB (11% difference vs. those not exposed). For UVTB5+, there was a 10% difference in willingness to further share knowledge gained on TB between participants who attended health education sessions and those not attending (Figure 1).

Knowledge-seeking behaviors were categorized as actions representing a deeper level of engagement with content, such as participation in more prolonged TB educational activities (whether virtual or face-to-face) including online review of TB knowledge content, playing online informational games, taking disease knowledge quizzes, direct messaging, episode viewing, attending lectures, talks, and storytelling. The included initiatives documented more than 800,000 individuals undertaking knowledge-seeking activities. Overall, 0.7% of all initiative engagements translated into knowledge-seeking behaviors, with variation across channels (digital-first campaigns varying from 0.2% for “Be the Change” and MTV Nishedh to 2.7% for TB Warriors—and onsite-first campaigns targeting smaller distinct populations achieving 23 and 72%, respectively for MumbraTB-ACTS and UVTB5+).

Digital engagement data showed positive increases in search engine key TB terms searches for “Be the Change” (14% increase) and TB Warriors (20% increase)—this is highly encouraging given the external benchmark of only one in four initiatives achieving search volume increases (35).

### 3.4 Healthcare-seeking behavior

The definition used for healthcare-seeking behavior (in contrast with either engagement and knowledge-seeking behavior) focused on tracking core indicators of intent to act toward presentation to care, such as calling national TB helplines and carrying out online searches for the closest TB clinic to present to care.

Across the India campaigns, more than 12,000 individuals accessed the National TB Helpline. Additionally, in “Be the Change” more than 105,000 government self-screening app installations were recorded—although the initiative tracked neither the subsequent impact of how many actual self-screening actions took place, nor follow-on enrollment in government database for presumptive TB. TB Warriors was associated with 7,215 clinic searches.

**Figure 2:**
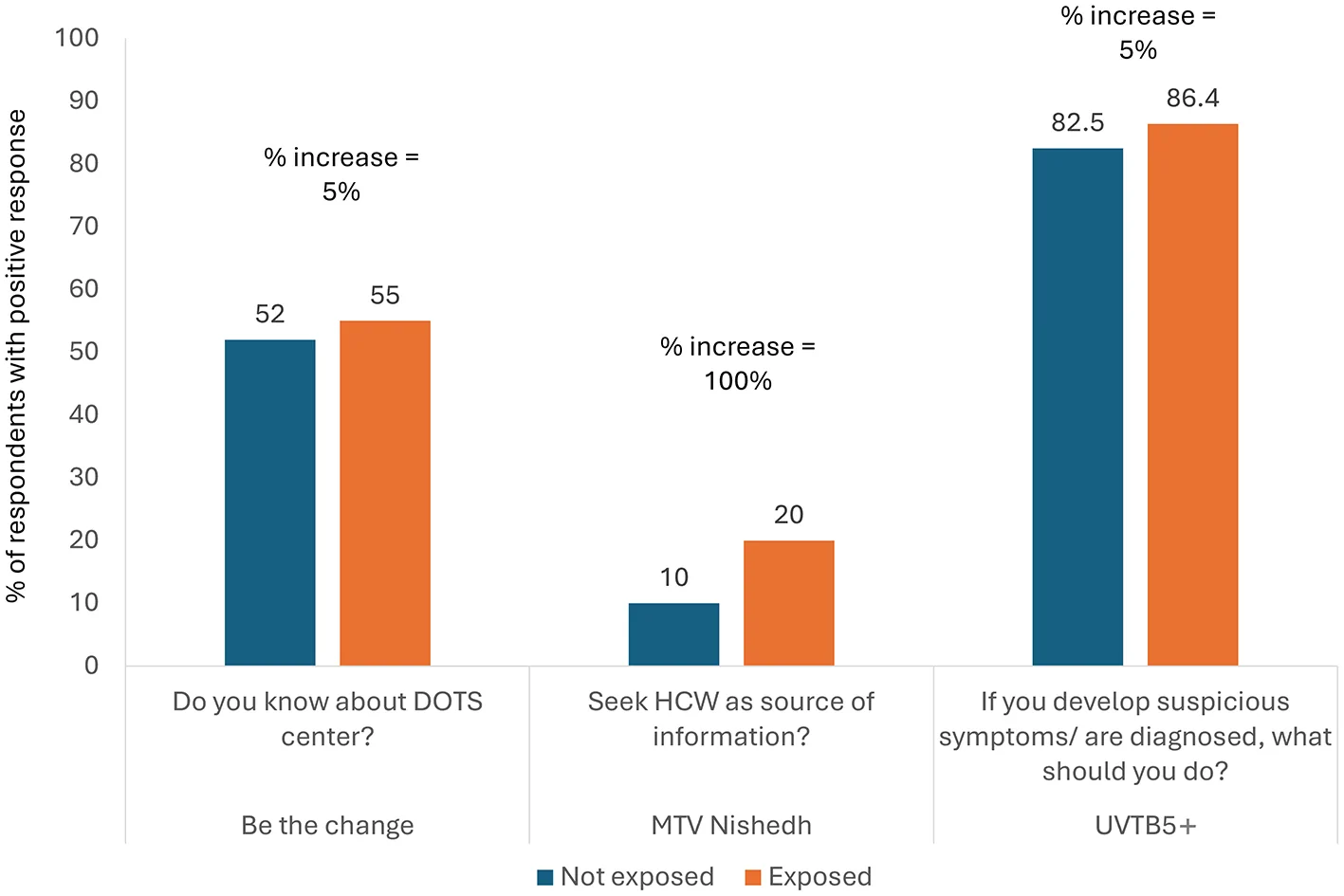
Healthcare-seeking knowledge. Responses on plan of action in case of TB symptoms in not exposed and exposed populations in Be the Change, MTV Nishedh, and UVTB5+. DOTS, Directly Observed Treatment Short-Course; HCW, healthcare worker; TB, tuberculosis.

Attitude-related information (as part of TB KAP) on healthcare-seeking behavior (Figure 2) was captured through survey responses in “Be the Change”, MTV Nishedh, and UVTB5+. Wide variation in awareness/willingness to tap into available resources was observed across the two initiatives from India −55% knew of DOTS treatment center availability in “Be the Change” post-intervention (6% increase), while exposure to the MTV Nishedh initiative was associated with 20% participants indicating that they would approach a HCW for guidance vs. 10% for non-exposed individuals. In contrast, UVTB5+ participants in China had a very high level of resource availability (86% of those receiving health education and just 5% change from those not receiving health education). The willingness to get tested for TB improved through intervention both in MTV Nishedh (63% for exposed vs. 43% for non-exposed; 47% change), and in UVTB5+ (91% for those receiving health education vs. 81% for those not receiving; 12% change).

### 3.5 Volunteer engagement

**Figure 3:**
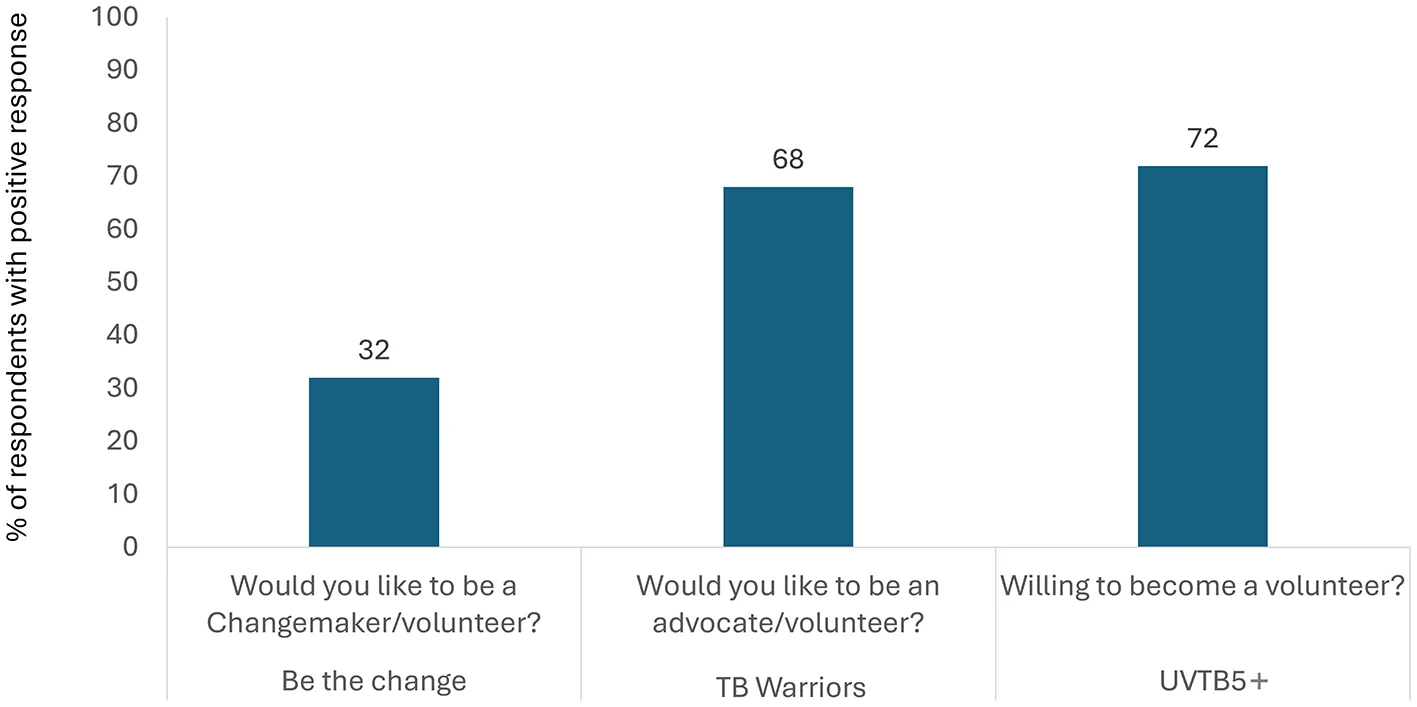
Volunteering interest among initiative target populations (KAP survey %) in Be the Change (*n* = 35,938), TB Warriors (*n* = 3,933), and UVTB5+ (*n* = 11,309). KAP, knowledge, attitudes, and practices; TB, tuberculosis.

“Be the Change,” TB Warriors, and UVTB5+ successfully recruited volunteers from their target populations (35,938, 3,933, and 11,309 respectively) while 32%, 68%, and 72% stated their willingness and interest to volunteer as part of survey responses (Figure 3). Both onsite, peer-to-peer campaign components executed on campus, as part of TB Warriors and UVTB5+, directly harnessed the energy of youth volunteering to contribute to the overall initiative impact.

The volunteering intent survey done by “Be the Change” reached 13.3 million youth through the Google DV360 platform, of which (3%) responded to the survey. A total of 71,535 youth showed positive intent to engage in TB initiatives, with 7,845 respondents identified as high-volunteering intent i.e., those most likely to engage in activities related to TB advocacy and community. Overall, 85% of high-intent youth were aware of TB as a disease, while 55% had previous volunteering experience. Self-satisfaction and passion (40%), followed by new skills development (35%) were top motivators, while lack of time (41%) was the biggest barrier to participation. Of note, most volunteers in “Be the Change” belonged to those in the lower 50% household income group.

### 3.6 Screening and diagnosis

Four out of five initiatives had screening components. Two measured the extent of digital self-screening (MumbraTB-ACTS and TB Warriors). Three had onsite screening with additional investment (MumbraTB-ACTS and TB Warriors had active case finding and UVTB5+ had intensified case finding). MumbraTB-ACTS demonstrated the ability of a poor urban population within an Indian slum community to utilize digital self-screening (157 individuals, representing 0.1% of a total 180,930 individuals screened), while TB Warriors had inversely proportional dynamics (18,358 individuals representing 93% of the total screened undertook digital self-screening).

Within the onsite campus campaign component, screening was undertaken for 1,346 (20%) of TB Warriors educational talk attendees, of whom 10% were referred to clinic (only < 5% actually confirmed attending the clinic). As part of UVTB5+, screening was undertaken for 617,025 (99%) of enrolling Sichuan students, of whom 112 were diagnosed and 330 started preventive therapy. Case notification for MumbraTB-ACTS increased during Q1–Q3 2022 compared with the same period during the previous year (1,088 vs. 736, respectively). Furthermore, MumbraTB-ACTS tracked completion of the patient journey beyond presentation to care, with 235 of 248 diagnosed patients (~95%) initiating treatment as part of the government pathway.

## 4 Discussion

The study aimed to document and evaluate innovative strategies addressing the TB healthcare-seeking delays in high-burden geographies within India, Indonesia, and China. The programs utilized either fully digital or hybrid (online/onsite) approaches to provide individuals with the opportunity to undertake knowledge seeking and improve their knowledge, attitudes, and behavior regarding TB care seeking. The programs aimed to inspire participants to initiate TB healthcare seeking and ultimately complete their presentation to care journey through to screening and diagnosis. The initiatives leveraged collaborations with creative content developers and media advertising agencies to amplifying learnings from digital health promotion impact achieved in other disease areas (36–38). The impact achieved is a direct reflection of the existing need in these geographies and target audiences.

In terms of impact on reach and engagement, all five initiatives were successful in interacting with their target populations, with each also demonstrating the effectiveness of strategies chosen within their local context to engage young people, resulting in more than 200 million youth being collectively reached and 100 million engaged. Onsite initiatives achieved their reach goals, and digital initiatives significantly exceeded these. Extent of reach is a direct function of available resources, target population size, communication media used, and length of campaign period. Hence, initiatives using digital mass-communication were expected to reach more people than initiatives with more resource-intensive hybrid online/onsite activities (e.g., posters, information stands, face-to-face group engagements). Of note, high-reach campaigns targeting a wide demographic might sometimes have lower engagement than more intensive targeted lower-reach campaigns (39), this was not necessarily the case in initiatives included here, emphasizing the importance of utilizing messaging and channels that promote direct interaction with the campaign, and therefore increase engagement and resulting TB-related behaviors, such as a TB screening. Program coordinators can also amplify the impact of an initiative through efforts to directly link participants with the next steps in the TB screening and diagnosis cascade. For example, MTV Nishedh Season 2 cross-promoted “Be the Change,” resulting in positive audience spillover (where viewers are invited to watch and engage with subsequent content).

Rather than choosing between media channels (e.g., between television in India, which represents the country's largest media platform, and digital in India), the focus should be on complementary availability (using and re-using the same content across multiple channels) (40, 41). More creative and bolder content leads to more (online) conversations, and this brings new audiences through influencer involvement (42, 43). The “power” of the messengers' voices [especially celebrities, movies stars, pop culture icons (44)], directly impacts engagement levels. The continued availability of online content means that the reach (and engagement) of an initiative is not limited by a campaign timeframe and allows for cumulative effect. A further key channel of communication available for impactful digital health promotion, that was not utilized in these initiatives, is the healthcare workers themselves, especially community health workers who are already connected digitally to the vulnerable populations they serve and, as such, could amplify messaging of future initiatives.

Concerns that populations in lower socioeconomic strata may not be reached adequately through mass media, including radio (1, 45), were partially addressed in “Be the Change” and TB Warriors, as both successfully activated the lower fiftieth household income percentile in urban areas. This stratum was also fully addressed in MumbraTB-ACTS, which solely focused on an urban slum geography. For initiatives with a hybrid online/onsite format, the quality of engagement resulting from creative two-way, peer-to-peer, volunteer-led interactions cannot be underestimated, particularly regarding addressing delays in TB healthcare seeking and care linkages (46).

All KAP surveys were conducted digitally or through computer-assisted telephone interviewing, enabling process simplification, alongside cost and time efficiencies. Disease transmission and awareness of supportive resources were identified as some of the biggest knowledge gaps, consistent with the United Nations' recent call to intensify TB awareness (47). Considering the impact on knowledge-seeking behavior, among the most significant changes was the increase in knowledge of TB transmission achieved with MTV Nishedh (40% non-exposed to 74% exposed; 84% change) and a 26% change in knowledge that TB is curable associated with UVTB5+ health education.

Discriminatory attitudes were not consistently measured and the comfort level in engaging with TB patients post-intervention varied widely from 16% in MTV Nishedh Season 2 (with a surprising increase vs. baseline) to 89% in UVTB5+. None of these campaigns undertaking a multi-channel approach measured differences in messaging effectiveness across messengers (e.g., whether celebrity involvement helps reduce stigma or which media channel engages most effectively). Furthermore, follow-through to presentation for care—following changes in healthcare-seeking behavior—was not assessed across initiatives. This represents a key gap for future study design, as understanding the real-world impact of behavior change (e.g., presentation to care after self-screening or database enrollment for presumptive TB) is essential to evaluating the effectiveness of TB initiatives such as those presented herein.

Previous studies have found that providing general information about TB and its treatment may not translate into change in behaviors (48). It is important that a campaign has a clear call to action that is communicated in a way that overcomes literacy and educational-level barriers, and that KAP assessment is directly linked to the campaign's call to action (49). It should be noted, however, that based on available data no causal relationship could be established between student attendance at onsite campus screening booths (e.g., those featured in MTV Nishedh, TB Warriors, and UVTB5+ campaigns) and engagement with the associated digital platforms. An additional consideration is whether a campaign can realistically achieve an impact on multiple KAP questionnaire items (which is often the ambition in TB health promotion) or whether single KAP items should be prioritized. Therefore, we believe that there is significant value in carrying out standardized pre-intervention surveys that facilitate understanding of knowledge gaps and identification of appropriate messaging. This can be further optimized (as was the case with initiatives such as “Be the Change” and TB Warriors) by extensive market research (both primary as well as secondary) into the target population's existing beliefs including via intent surveys. Nonetheless, the measured KAP levels here and elsewhere signal that action is needed and that such interventions can have impact (50, 51).

As per theoretical change models, knowledge-seeking behavior is likely to enable subsequent healthcare-seeking behavior (52). Very few studies have reported the impact of KAP change and knowledge-seeking behavior on healthcare-seeking behavior (9, 18), especially following through to presentation to care. Across the initiatives, the ratio of documented knowledge seeking vs. healthcare seeking is approximately a ratio of 7:1. This is a function of measurement frameworks utilized and not necessarily an indicator of reality (it is possible that some health-seeking actions were not captured given the challenges in tracking a population acting outside of their local health system).

Where in-scope initiatives directed individuals to undertake (self-)screening and linked them to government provision of care, hybrid online/onsite approaches (which included an active case-finding component at higher investment) were able to also measure direct follow-on impact. Indeed, on-the-ground screening results for MumbraTB-ACTS and UVTB5+ revealed an impact on diagnoses. Under MumbraTB-ACTS, of the 180,930 screened individuals 248 were diagnosed (of whom 235 started treatment); it should also be noted that case findings increased by ~48% compared with the previous year. Under UVTB5+ (Sichuan site only), 617,000 individuals were screened, 112 were diagnosed, and an additional 330 started preventive therapy. Additionally, of the 1,346 individuals screened on campus under TB Warriors, 10% were referred to the health clinic. Although fewer than 5% of those referred presented to the clinic, it is possible that more students went to the clinic after the close of the reporting period. Unfortunately, case-finding data are not available for any of the online screening components.

The importance of multisector collaboration cannot be understated from a multiplier effect perspective—on both surrounding communities and healthcare workers from the same geography as target populations (both are also exposed to the same messaging and calls to action). Future research should measure the magnitude of KAP change across all these different segments to determine whether there is a synergistic impact on utilization of screening and referral pathways.

Volunteering in TB, especially for health promotion (as part of broader advocacy) represents an untapped potential pool of resources to address health inequities and accelerate change (53). Social media has proven to be a powerful tool for supporting such efforts and therefore puts youth in a unique situation to have a major contribution (54). The results from the “Be the Change” volunteering intent survey were informative in terms of both the proportion and scale of numbers of youth likely to volunteer, as well as their motivators and barriers in this respect. Close to 36,000 individuals were ultimately recruited within 3 months through the “Be the Change” social media campaign, as the campaign transitioned into a youth movement. Self-satisfaction, passion, and the development of new skills were identified as the top motivators for volunteering, with lack of time the most common barrier. That most volunteers in “Be the Change” came from the lower 50% of household income illustrates a desire of youth from this impacted setting to directly help their communities. While data are scarce, the outputs of such a recruitment drive compared favorably with multi-year TB volunteering campaigns that have averaged a cumulative of 15,000 volunteers (55–57).

Leveraging young people as powerful agents of change proved effective across the initiatives, suggesting that sustained efforts should be made to activate and empower young changemakers to carry out health promotion to end TB globally (29). UVTB5+ activity impact was a direct function of mobilizing youth, similar to the dynamics observed in the TB Warriors onsite campaign component. In addition to online initiatives, university campuses are ideal locations for implementing youth activation initiatives, given the high concentration of motivated volunteers that exist in campus environments (58, 59).

Although numerous efforts are underway within health systems to activate proactive TB behaviors at the community level and early in the individual care pathway, there remains a lack of specific normative guidance on best practices and the minimum requirements for assessing the impact of such interventions. This gap persists despite the presence of diverse strategies outlined under Advocacy, Communication, and Social Mobilization (ACSM) and Social and Behavior Change Communication (SBCC) models and is further supported by the diversity of metrics evaluated across the initiatives explored herein. In our analysis, we attempted to use similar or consistent definitions for outcomes, such as reach and engagement, plus indicators for future presentation to care (knowledge-seeking and healthcare-seeking behaviors). While reach is relatively straightforward to define, engagement is a more tenuous concept since different behaviors can represent different levels of commitment by the participant toward the ultimate goal of presentation to care, resulting in variable definitions in program metrics.

Therefore, there is a critical need for a framework that incorporates standardized operational guidance on best practices and for intervention impact measurement at the TB healthcare-seeking stage, prior to entering the health system (60). Future program evaluations, ideally under a global framework, should incorporate systematic approaches to assess and rank outcomes across key dimensions such as acceptability, feasibility, accessibility, equity, person-centered preferences, and values, while aligning with the broader WHO classification of self-care interventions for health.

It will be key to develop standardized healthcare seeking outcome definitions and indicators, as well as a minimum core set of KAP questions for incorporation into pre- and post-survey measurement tools. Providing optimal target levels for indicators (further segmented by population type and geographic setting) would enable tracking of impact evolution over time as well as cross-comparisons. To further illustrate the need for normative guidance, the interrelated nature of indicators must be considered. Interventions should not measure “before” indicators without corresponding “after” measurement of the same indicator, nor should they focus solely on reach without assessing the effect on healthcare-seeking behaviors and measurable real-world actions (e.g., increased presentation for TB testing in specific geographies).

These behavioral indicators should be complemented by disease-level outcomes, including changes in diagnosis rates, while acknowledging the challenges of attribution, delayed impact, and potentially weak correlations inherent in such interventions. It would also be of value to clarify through guidance (and capacity building) the responsibilities of healthcare workers and the health system as it relates to the leadership and support of such health promotion campaigns, as part of providing quality accessible care (42). Overall, defining such a framework could significantly support health systems and health workers in their provision of equitable access to quality care and implementation of TB initiatives intervening at early stages of the patient journey. Additionally, future programs should consider incorporating resilience strategies to mitigate the impact of unforeseen disruptions. While the temporal overlap between our initiatives and the COVID-19 pandemic did not introduce unmanageable operational challenges, as they all took place after lockdowns were lifted, intervention design sought to enable simplified practical execution, limiting complex linkage measurement for integrated online-to-offline patient journeys.

As to limitations, there were difficulties in assessing cumulative outcomes from the five different initiatives given the variations in engagement definition and measurement indicators. In addition, there is inherent uncertainty within such real-world data (e.g., reporting biases and incomplete data). Furthermore, due to methodological and outcome-related heterogeneity across initiatives, alongside differences in baseline indices or comparators, reliable statistical comparisons of knowledge change could not be performed. To enable meaningful cross-initiative comparisons in the future, any general framework should clearly define standardized methodological components (e.g., survey questions).

Finally, it is important to further define the individual as well as synergistic effect that comes from enhancing patient-initiated pathways to diagnosis and/or undertaking provider-initiated pathways to diagnosis. As previously mentioned, both are needed to end the TB epidemic, however, it is possible that, over the long term, enabling a greater number of people to achieve optimized knowledge levels and proactively self-present to care may be more efficient than population-wide screening. Nevertheless, despite these limitations, these initiatives provide evidence on need for standardized operational guidance for such types of interventions.

## 5 Conclusion

This study reviewed and assessed five youth activation and education initiatives supported by the private sector, seeking to address delays in presentation to TB care. Given the scarcity of literature regarding effectiveness of youth activation programs in impacting TB healthcare seeking, these findings may be useful for future programmatic implementation efforts as well as for developing standardized operational guidance for the design and outcome measurement of TB healthcare-seeking programs.

A comprehensive understanding of youth personae across geographies, sex, household characteristics, etc. is a prerequisite for designing effective, large-scale (yet hyper-targeted), sustainable, digital, creative strategies that positively engage youth. We recommend using insight-informed, tailored, action-oriented, highly practical and empowering messaging, messengers that normalize conversations and create safe spaces as well as streamlined digital linkages to government systems—this should nurture an approach were people can self-screen and self-present. The success of any campaign will depend on how its efforts translate into early healthcare-seeking behavior and ultimately numbers screened (61).

To amplify the effects of youth activation initiatives in the future, health workers could play a vital role in transforming the pre-presentation to care experience through direct engagement with the mass youth volunteer movement. Integration of front-line health workers and the formal health system could create synergies resulting in sustainable initiatives that broaden population-wide case finding (62, 63) as part of the approach to eradicate TB. Young populations in high burden TB geographies are highly connected, energetic, and tech-savvy. With support from health workers, youth have the potential to lead health promotion efforts, thereby expanding the health workforce in a cost-effective and sustainable way.

## Data Availability

The raw data supporting the conclusions of this article will be made available by the authors, without undue reservation.
