# Supplementary material for: A synthesis of youth activation initiatives and their impact on tuberculosis knowledge, knowledge-seeking, and healthcare-seeking behavior in vulnerable populations
Source: Front Tuberc. 2026 Jan 9;3:1668711. doi: 10.3389/ftubr.2025.1668711 (PMC13494979; doi:10.3389/ftubr.2025.1668711)
Supplement: Supplementary file 1 [file Data_Sheet_1.docx]

# 1 Supplementary Material

## 2 Table S1. “Be the Change” intent survey and pre- and post-campaign knowledge survey.

| **Question** | **Answer options/categories** |
| --- | --- |
| **Intent survey** |  |
| 1. Are you aware about tuberculosis (TB) as a disease? | - Yes - No |
| 2. Are you aware that there are tuberculosis (TB) related programs undertaken by the government? | - No - Aware - Aware about such programs and aspire to participate - Aware and participated |
| 3. Have you volunteered in the past in any such programs undertaken by the government or private institutions? | - Yes - No |
| 4. Would you like to be the changemaker/volunteer for a national level program to impact a million lives of tuberculosis (TB) patients? | - Yes - No |
| If “Yes” to question 4: |  |
| 5. What would motivate you to volunteer? | - Certification/recognition - New skills development - Self-satisfaction and passion - None of the above |
| 6. What kind of volunteering activities would you be interested in? | - Online activation - Working with the community - Internship with the state/national government - Working with healthcare centers - All of the above |
| 7. How long would you want to stay signed up for the program? | - <3 months - 3 months - 6 months - 1 year |
| If “No” to question 4: |  |
| 8. What prevents you to join the cause? | - No time - No interest - Don’t believe in the purpose or the cause - Any other |
| **Knowledge survey** |  |
| 1. According to you, what is tuberculosis (TB)? | - Symptom of HIV infection - Hereditary disease only affecting children - Lung disease caused by smoking - Infectious disease affecting lungs |
| 2. How does tuberculosis (TB) spread? | - Water (e.g., sharing water with a TB infected person) - Food (e.g., sharing food with a TB infected person) - Air (e.g., TB infected person coughs or sneezes) - Touch (e.g., holding hands with an infected person) |
| 3. According to you, what is/are the symptoms of tuberculosis? | - Cough for more than 2 weeks - Fever - Weight loss - Blood in sputum |
| 4. Which of these samples are used for tuberculosis (TB) testing? | - Sputum (saliva) - Blood - Feces (stool) - Urine |
| 5. Tuberculosis (TB) is a curable disease. | - True - False |
| 6. Do you know about DOTS center? | - Yes - No |
| 7. According to you, does tuberculosis (TB) occur only in lower socioeconomic groups? | - Yes - No |
| 8. In which age group can tuberculosis (TB) occur? | - Children and adolescents <18 years - Adults (>18 years of age) - Any age group (both children and adults) - Not sure/don’t know |

An online pre-campaign survey targeted the best-performing cohorts from an initial intent survey and assessed their basic awareness of TB. Participants surveyed were those with the greatest intent to be involved (i.e., highest survey scores). The best performing cohorts (six) were: P1: Solution aware; people who are aware and have previously volunteered in such programs; P2: Active seeker; people who aren’t aware and have previously volunteered in such programs; P3: Problem aware; people who are aware and haven’t previously volunteered in such programs; one age group (aged 18–24 years), and two income levels (lower 50% household income level and top 10% household income level) with past volunteering experience and TB awareness.
DOTS, Directly Observed Treatment Short-Course; HIV, human immunodeficiency virus; TB, tuberculosis.

## 3 Table S2. MTV Nishedh baseline and endline surveys.

| **Question** | **Answer options** |
| --- | --- |
| 1. Sources of information about TB | - TV - Doctor/healthcare center - Family members - Friends - Social media - Online video platforms - Helpline numbers - Confidential chatbot - Don’t know/can’t say |
| 2. Major symptoms of TB | - Persistent cough - Coughing blood/mucus - Chest pain or pain while breathing/coughing - Fever - Fatigue - Sudden weight loss - Loss of appetite - Chills - Night sweats - Don’t know/can’t say |
| 3. Plan of action in case of symptoms of TB | - Get immediately tested for TB - Consult a general physician - Should completely isolate themselves from others - Start self-medication/home remedies - Consult an ayurvedic physician/healer - Consult a homeopathic physician |
| 4. TB spreads through air | - Strongly Agree - Agree - Disagree - Strongly Disagree - Don’t know/can't say |
| 5. TB is curable |  |
| 6. Treatment for TB is free in India |  |
| 7. TB is more prevalent in poorer sections of the society |  |
| 8. If I test positive for TB I would not want to share this information with others |  |
| 9. I would be uncomfortable to stay in touch with someone known to have TB |  |
| 10. It is important to share information about TB and its treatment |  |
| 11. Ever tested for TB owing to the symptoms | - No - Never had any such symptoms - Yes - I don’t want to discuss |
| 12. Care-seeking behavior in case of potential TB symptoms | - Get immediately tested for TB and seek treatment - Consult a traditional healer - Start self-medication/home remedies - Complete isolation from others - Will not take any action - Don’t know/can’t say |

TB, tuberculosis.

## 4 Table S3. TB Warriors 2.0 intent survey.

| **Question** | **Answer options/categories** |
| --- | --- |
| 1. Are you aware about tuberculosis (TB) as a disease? | - Yes - No |
| 2. Are you aware that there are tuberculosis (TB) related programs undertaken by the government? | - No - Aware - Aware about such programs and aspire to participate - Aware and participated |
| 3. Have you advocated in the past for any social cause? | - Yes - No |
| 4. Would you like to be the TB disease advocate (TB Warrior) who positively impacts a million lives of tuberculosis (TB) patients? | - Yes - No |
| If “Yes” to question 4: |  |
| 5. What would motivate you to be a TB Warrior? | - Certification/recognition - Self-satisfaction and passion - Community betterment - None of the above |
| 6. What kind of volunteering activities would you be interested in? | - Social media advocacy - Community focused activities - Working with TB healthcare centers - All of the above |
| If “No” to question 4: |  |
| 7. What prevents you to become a TB Warrior? | - No time - No interest - Don’t believe in the purpose or the cause - Any other |

Survey was conducted to understand the intent to become a TB Warrior (i.e., confirmed intent to become a TB advocate). Ideal candidates (who scored full marks on the survey) were top performing cohorts in the 18–24 years age group and a mix of lower 50% household income level and top 10% household income level.
TB, tuberculosis.

## 5 Table S4. UVTB5+ core knowledge survey.

| **Question** | **Answer categories** |
| --- | --- |
| 1. What is TB? | - Acute communicable disease - Acute non-communicable disease - Chronic communicable disease - Chronic non-communicable disease |
| 2. How is TB transmitted? | - No idea - Through respiratory tract transmission - Through digestive tract transmission - Through blood-borne transmission |
| 3. What are the suspicious symptoms of TB? | - No idea - Abdominal pain and diarrhea - Cough and sneeze - Cough, sputum for more than 2 weeks, or sputum with blood |
| 4. If you suspect that you have TB, what should you do? | - No idea - Visit local TB designated medical institutions - Visit a private clinic - Buy your own medicine |
| 5. Is TB curable? | - No idea - Most of them can be cured - All of them can be cured - Incurable |
| 6. While at school, when you develop suspicious symptoms of TB or are diagnosed with TB, what should you do? | - Take lessons while in therapy - Immediate hospitalization - Observation without treatment if symptoms are not severe - Take the initiative to report to the school and do not conceal your illness or bring it to class |
| 7. What practice is beneficial in preventing the spread of TB? | - Not drinking raw water - No littering - Regular diet - Open windows regularly for ventilation |
| 8. Which lifestyle habit will improve your immunity? | - Stay up late, overeat, and exercise occasionally - Stay up late, irregular diet, lack of exercise - Wear a mask, eat a balanced diet, and exercise consistently - Keep early hours, balanced diet, and consistent exercise |

TB, tuberculosis.
